# Changes in Global Gene Expression Associated with 3D Structure of Tumors: An *Ex Vivo* Matrix-Free Mesothelioma Spheroid Model

**DOI:** 10.1371/journal.pone.0039556

**Published:** 2012-06-21

**Authors:** Heungnam Kim, Yen Phung, Mitchell Ho

**Affiliations:** Laboratory of Molecular Biology, National Cancer Institute, National Institutes of Health, Bethesda, Maryland, United States of America; Roswell Park Cancer Institute, United States of America

## Abstract

Tumor microenvironments present significant barriers to anti-tumor agents. Molecules involved in multicellular tumor microenvironments, however, are difficult to study ex vivo. Here, we generated a matrix-free tumor spheroid model using the NCI-H226 mesothelioma cell line and compared the gene expression profiles of spheroids and monolayers using microarray analysis. Microarray analysis revealed that 142 probe sets were differentially expressed between tumor spheroids and monolayers. Gene ontology analysis revealed that upregulated genes were primarily related to immune response, wound response, lymphocyte stimulation and response to cytokine stimulation, whereas downregulated genes were primarily associated with apoptosis. Among the 142 genes, 27 are located in the membrane and related to biologic processes of cellular movement, cell-to-cell signaling, cellular growth and proliferation and morphology. Western blot analysis validated elevation of MMP2, BAFF/BLyS/TNFSF13B, RANTES/CCL5 and TNFAIP6/TSG-6 protein expression in spheroids as compared to monolayers. Thus, we have reported the first large scale comparison of the transcriptional profiles using an ex vivo matrix-free spheroid model to identify genes specific to the three-dimensional biological structure of tumors. The method described here can be used for gene expression profiling of tumors other than mesothelioma.

## Introduction

Most human cells are located in and around blood vessels. It is this architecture that facilitates the delivery of oxygen and nutrients to the cells and allows for the efficient delivery of drugs [1], [2]. The proliferation of solid tumor cells forces blood vessels apart, creating a population of cells distant from vessels. The resultant tumor microenvironment is recognized as a hallmark of drug resistance found in solid tumors with studies showing that drugs can usually penetrate a few cell diameters from blood vessels into the tumor tissue [1].

The 3D spheroid model has emerged as the method of choice to study many aspects of malignant cell behavior *ex vivo* [3], [4]. Spheroids reflect many important properties of solid tumors, including the development of an extracellular matrix (ECM), cellular junctions between epithelial cells, and gradients of nutrient concentration and cell proliferation from the exterior to the center. Spheroids can develop central necrosis and regions of hypoxia. Interestingly, Bissell and colleagues for the first time found that all signal transduction pathways were integrated reciprocally in 3D laminin-rich ECM on-top cultures of breast cancer while these pathways appear to be disconnected on 2D monolayer tissue culture [5]. *Ex vivo* 3D culture of many other tumor types (including mesothelioma) has not been evaluated by global gene expression profiling. The role of a laminin-rich artificial ECM in both *in vitro* and *in vivo* tumor progression and morphology is well-documented [6], [7], [8]. It is not clear whether an artificial ECM plays a role in the expression profiling of 3D tumor culture. To address this issue, we think it is important to evaluate global gene expression profiling without artificial ECM.

Here, we performed large global gene expression profiling using an *ex vivo* matrix-free tumor model. In this way, we focused on gene expression profiling related to the 3D structure of tumors independent of artificial ECM. We chose NCI-H226 cells as the cell model for mesothelioma in the present study given that the cell line is (*a*) one of the NCI-60 cancer cell models (http://dtp.nci.nih.gov/docs/misc/common_files/cell_list.html), and (*b*) widely used in mesothelioma research as cell and preclinical tumor models [3], [9].

## Materials and Methods

### Cell line

The human mesothelioma cell line, NCI-H226, was obtained from the American Type Culture Collection (ATCC; Rockville, MD). The cell line was maintained as adherent monolayer cultures in RPMI 1640 medium (Invitrogen, Carlsbad, CA) supplemented with 10% fetal bovine serum (HyClone, Logan, UT), L-glutamine, pyruvate, nonessential amino acids, and penicillin-streptomycin (Invitrogen, Carlsbad, CA) and incubated in 5% CO_2_ with balance of air at 37°C. Cells were seeded at 2×10^5^/mL in T-75 tissue culture flasks (Corning Incorporated, Acton, MA). Cells were harvested and the media were changed twice a week. Cells were confirmed to be negative for mycoplasma.

### Spheroid formation

We made *ex vivo* tumor spheroids following the protocol previously described [3], [10]. A 96-well Greiner suspension culture plate (Sigma, St. Louis, MO) was coated with 50 µL of 5 mg/mL of poly-HEMA (poly-2-hydroxyethyl methacrylate; Sigma-Aldrich) in 95% ethanol and evaporated with lid on at room temperature for 72 hours. Mesothelioma cells were grown to near confluency and dissociated into single cells with Accutase (BD Biosciences, San Jose, CA). Each well contained 10,000 cells for one spheroid. The plate was then centrifuged at 1000 rpm for 10 minutes to initiate cell-cell interaction and incubated at 37°C, 5% CO_2_ for 24 hours. The spheroids are stable for 48–72 hours and can be easily transferred using a regular pipette without dissociating.

### Microarray analysis

Microarray analysis experiments were performed in triplicates. A total of six samples (three for monolayers and three for spheroids) were analyzed. Total RNA was prepared from NCI-H226 spheroid and monolayer cells using RNeasy Plus Mini Kit according to the manufacturer's instructions (Qiagen, Valencia, CA). Eight micrograms of total RNA was amplified using the one-cycle amplification method (Affymetrix, Santa Clara, CA). Twenty micrograms of aliquot of labeled cRNA was fragmented by heat and ion-mediated hydrolysis, and hybridized to a Human Genome U133A Plus 2.0 Array (47,000 transcripts and variants including 38,500 human genes; SAIC-Frederick, Frederick, MD). The arrays were washed and stained in a Fluidics Station 450 and scanned using a GeneChip Scanner 3000 (Affymetrix, Santa Clara, CA) and GeneChip Operating Software (Affymetrix). Expression values for each sample from microarray were normalized to median expression values. Statistical analyses including a student's *t*-test was performed using Genespring GX 7.3 software (Santa Clara, CA). *p* values less than 0.05 were considered significant. Hierarchical clustering was used to group entities and conditions based on the similarity of expression profiles. The raw data has been deposited at the National Center for Biotechnology Information Gene Expression omnibus (GEO) with accession number GSE37629.

### Gene ontology analysis

One hundred forty two transcripts with a five or greater fold change in expression as well as a student's *t*-test output of <0.05 were used to identify significant differential expression. Gene ontology enrichment analysis was performed using the Database for Annotation, Visualization and Integrated Discovery (DAVID) functional annotation tool (http://david.abcc.ncifcrf.gov). Twenty seven membrane expressing genes out of 142 transcripts were used to generate biological function using Ingenuity Pathways Analysis (IPA; www.ingenuity.com, Ingenuity Systems, Redwood City, CA). Gene accession numbers were imported into the Ingenuity Pathway Analysis version 3.1 software, and gene products were categorized based on location, cellular components, and reported or suggested biological functions using the software. Mapping to genetic networks available in the Ingenuity database was also performed with ranking by score, the probability that a collection of genes equal to or greater than the number in a network could be achieved by chance alone. A score of three indicates that there is a 1/1000 chance that the focus genes are in a network due to random chance. Therefore, scores of three or higher have a 99.9% confidence level of not being generated by random chance alone. This score was used as the cut-off for identifying gene networks.

### Western blot analysis

NCI-H226 cells were allowed to grow for 48–72 hours. After seeding until approximately 60% confluent, 2×10^6^ monolayer cells were centrifuged and collected, washed with 1 mL PBS and resuspended in 100 µl of immunoprecipitation assay buffer containing 2% SDS and protease inhibitors (“Complete Mini-EDTA Free” protease inhibitor tablet, Roche, Mannheim, Germany) to lyse cells or spheroids. Four cycles of freezing at −80°C and thawing at 37°C were repeated. Protein lysate was centrifuged at 10,000 rpm for 1 minute, supernatant was collected and protein concentration was measured via Coomassie Plus Protein Assay (Thermo Scientific/Pierce, Rockford, IL). Samples containing 50 µg of cell lysate per lane were separated by SDS-PAGE, transferred onto PVDF membranes, and incubated with primary antibodies. The primary antibodies used include polyclonal anti-MMP2 (Santa Cruz Biotechnology, Inc., Santa Cruz, CA), polyclonal anti-TSG-6 (Santa Cruz Biotechnology, Inc.), polyclonal anti-RANTES (Santa Cruz Biotechnology, Inc.) and monoclonal anti-BAFF (R&D Systems, Inc., Minneapolis, MN). Primary antibodies were detected by secondary goat anti-rabbit antibodies or anti-mouse antibodies conjugated with horseradish peroxidase (Jackson ImmunoResearch Laboratories, Inc., West Grove, PA). Signals were visualized by an enhanced luminol-based chemiluminescence Western blotting detection kit (GE Healthcare, Piscataway, NJ).

## Results

### Global gene expressing profiling of 3D mesothelioma spheroids

**Figure 1:**
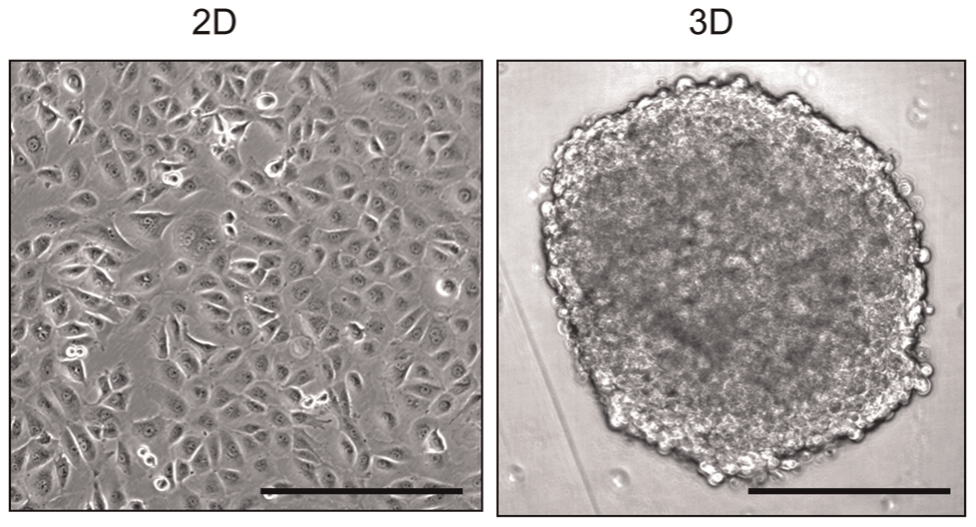
The *ex vivo* tumor spheroid model. Microscopic images of monolayers and spheroids of human mesotheliomoa cell line NCI-H226. 2D: monolayers; 3D: spheroids. Scale: 400 μm.

To identify genes specific to the 3D structure of tumors, we cultured mesothelioma spheroids using the NCI-H226 cell line and compared the global gene expression profiles of NCI-H226 spheroids and monolayers using the Affymetrix Human Genome U133A Plus 2.0 GeneChip microarrays. We used the NCI-H226 mesothelioma cell line, which was originally isolated from the pleural fluid of a malignant mesothelioma patient, and may also be used to grow clinically relevant mesothelioma in mice [9]. We cultured each spheroid from 10,000 cells. After 24 hours of incubation, we found the formation of tight spheroid disks with smooth edges which appeared to be uniform with a diameter of approximately 700 µm with a thickness of 150 µm (Fig. 1). The expression of general mesothelioma markers (mesothelin, cytokeratin 5/6, calretinin, HBME-1, thrombomodulin, and WT-1) in spheroids is consistent with those of mesothelioma specimens [3]. Unlike a previous study using breast cancer cells [5], our spheroid model does not use any matrix [3], [10]. The formation of NCI-H226 spheroids is stably intact. A statistical summary for the differentially expressed probe sets between spheroids and monolayers is shown in Table 1. Out of 54,675 total probe sets, 1,808 probe sets including 639 upregulated probe sets and 1,115 downregulated probe sets were significantly different between the two groups (*p*<0.05) (Table 1).

**Table 1:** Statistics for microarray with a *p* value <0.05.

Fold Changes	Number of Probe sets	
>2	901	Up: 397
		Down: 504
>3	368	Up: 220
		Down: 148
>5	142	Up: 112
		Down: 30

Gene expression analysis was performed using the Affymetrix Human Genome U133 Plus 2.0 GeneChip Array (54,675 probe sets). A statistical analysis was performed using Genespring GX 7.3 software. Data used with a *p* value <0.05. *p*-value computation was performed using asymptotic, multiple testing correction (Benjamini-Hochberg).

**Figure 2:**
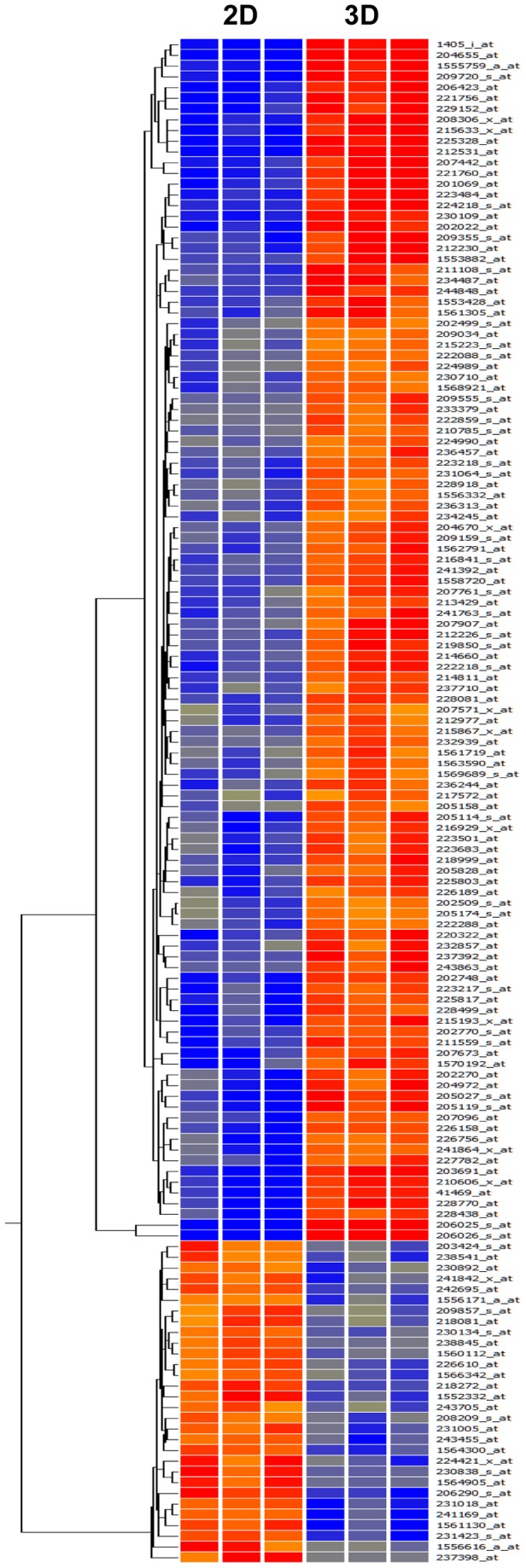
Hierarchical clustering of microarray data. Differentially expressed 142 probe sets with a five or greater fold change in expression as well as a student's *t*-test output of *p*<0.05 were visualized by Genespring program. Red indicates higher expression of the gene in NCI-H226 spheroid cells compared with monolayer cells (up-regulated), and green indicates lower expression of the gene compared with the NCI-H226 monolayer cells (down-regulation). Each row represents the expression level for the transcripts tested for an individual sample. Each column shows the expression levels for a single transcript tested for different samples. The entire list of 142 probes is presented in Table S1. 2D: monolayers; 3D: spheroids.

In this analysis, 142 probe sets (112 upregulated probe sets and 30 downregulated probe sets) in NCI-H226 spheroids compared to monolayers exhibited at least a five fold-change in expression levels and a *p*-value <0.05 (Table S1). The significantly changed 142 probes were used for hierarchical clustering and gene ontology analysis. As shown in Figure 2, hierarchical cluster analysis of significantly differentially expressed genes successfully segregated NCI-H226 spheroids and monolayers. Samples with similar patterns of expression of the genes studied were clustered together, as indicated by the dendrogram. The hierarchical clustering of the 142 genes that were most differentially expressed in the NCI-H226 spheroid cells versus the H226 monolayer cells is illustrated.

### Functional analysis of differential genes

**Figure 3:**
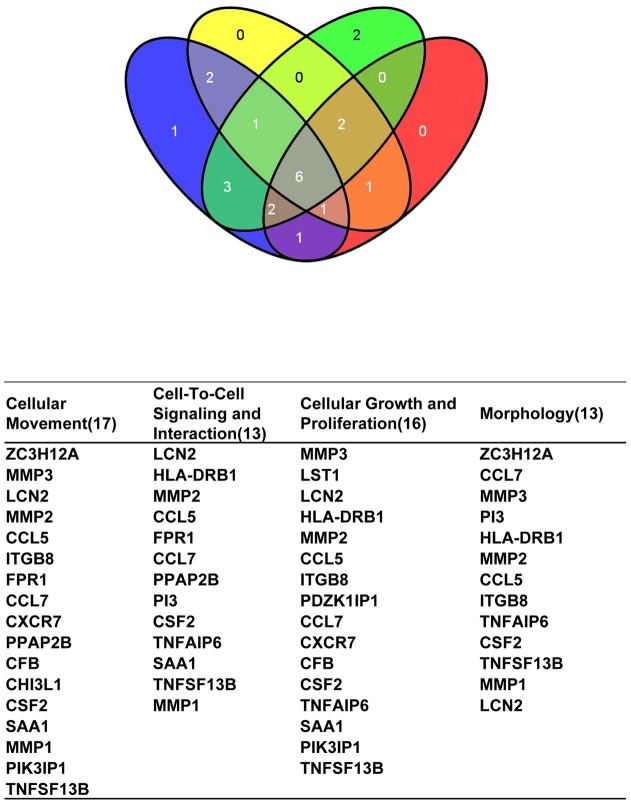
Venn diagram representing the number of transcripts related to biological functions, cellular movement, cell-to-cell signaling, cellular growth and proliferation and morphology for spheroids (3D) compared to monolayer (2D) NCI-H226 cells. Venn diagram incorporates 142 transcripts that have a *p*<0.05 and at minimum a five-fold change between NCI-H226 spheroids and monolayer cells. Blue indicates 11 genes for cellular movement, yellow indicates 13 genes for cell-to-cell signaling and interaction, green indicates 16 genes for cellular growth and proliferation, and red indicates 13 morphology-related genes.

To gain insight into the biological significance of the 142 probe sets identified in our microarray analysis, we interrogated the gene list for enrichment of known biological categories and performed Database for Annotation, Visualization and Integrated Discovery (DAVID) gene ontology analysis (Table 2). DAVID gene ontology determined the functional categories of significantly affected genes. DAVID analysis indicated that upregulated genes primarily affected immune response, wound response, lymphocyte stimulation and response to cytokine stimulation, whereas downregulated genes were primarily associated with apoptosis (SOD2, SPHK2 and CIAPIN1). Table 3 shows 27 genes differentially expressed and associated with cell membrane between NCI-H226 spheroids and monolayers. We also performed gene ontology analysis using the Ingenuity Pathway Analysis tool, which yielded 17 genes that were identified as biologic processes of cellular movement associated genes, 13 genes that were associated with cell-to cell signaling and 16 genes that were related with cellular growth and proliferation and morphology (Table 4). A Venn diagram using genes in those biological categories shows the identification of common genes (including MMP2, CCL5, TNFSF13B CCL7, CSF2 and LCN2) involved in the biologic processes of cellular movement, cell-to-cell signaling, cellular growth and proliferation and morphology (Figure 3). CCL5 and TNFSF13B are also known as RANTES and BAFF/BLyS, respectively.

**Table 2:** The gene ontology (GO) identified by functional analyses.

Up-Regulation			
Go Term	*P* Value	Fold Enrichment	Genes
GO:0006955: Immune response	2.00E−04	3.90E+00	CSF3, GBP2, HLA-DRB1 /// HLA-DRB3 /// HLA-DRB4, OAS2, TNFSF14, CCHLA-DRB1 /// HLA-DRB4, 208306_X_AT, TNFSF13B, CCL3 /// CCL3L1 /// CCL3L3 /// LOC728830, IL1F9, GBP1, CCL5, LST1
GO:0009611: Response to wounding	2.90E−02	2.90E+00	SOD2, CD36, CCL5, NFKBIZ, TNFAIP6, SAA4, NFKBIZ, CCL3 /// CCL3L1 /// CCL3L3 /// LOC728830
GO:0031294: Lymphocyte costimulation	3.50E−02	5.50E+01	TNFSF14, TNFSF13B
GO:0031295: T cell costimulation	3.50E−02	5.50E+01	TNFSF14, TNFSF13B
GO:0034097: Response to cytokine stimulus	4.80E−02	8.40E+00	MMP3, CCL5, CDKN2B

Down-Regulation			
Go Term	*P* Value	Fold Enrichment	Genes
GO:0006916: Anti-apoptosis	2.00E−02	13.13	SOD2, SPHK2, CIAPIN1
GO:0042981: Regulation of apoptosis	5.00E−02	4.49	SOD2, SPHK2, 237398_AT, CIAPIN1
GO:0043067: Regulation of programmed cell death	5.00E−02	4.44	SOD2, SPHK2, 237398_AT, CIAPIN1
GO:0010941: Regulation of cell death	5.00E−02	4.43	SOD2, SPHK2, 237398_AT, CIAPIN1

Gene ontology was performed using the Database for Annotation, Visualization and Integrated Discovery (DAVID) that were overrepresented (*p* value <0.01) in our list of 142 significantly differentially expressed transcripts (112 up and 30 down) in spheroids compared to monolayers of NCI-H226 cells. Biological process was used as a category of gene ontology term for this analysis.

**Table 3:** Genes differentially expressed in cell membrane between NCI-H226 spheroids and monolayers.

Fold Changes	ID	Symbol	Entrez Gene Name
66.8	206026_s_at	TNFAIP6	Tumor necrosis factor, alpha-induced protein 6
39.9	209395_at	CHI3L1	Chitinase 3-like 1 (cartilage glycoprotein-39)
20.9	1405_i_at	CCL5	Chemokine (C-C motif) ligand 5
16.5	212531_at	LCN2	Lipocalin 2
12.9	41469_at	PI3	Peptidase inhibitor 3, skin-derived
10.8	204475_at	MMP1	Matrix metallopeptidase 1 (interstitial collagenase)
10.4	202357_s_at	CFB	Complement factor B
10.1	212230_at	PPAP2B	Phosphatidic acid phosphatase type 2B
8.9	223502_s_at	TNFSF13B	Tumor necrosis factor (ligand) superfamily, member 13b
8.4	201069_at	MMP2	Matrix metallopeptidase 2 (gelatinase A, 72kDa gelatinase, 72kDa type IV collagenase)
7.7	229152_at	C4orf7	Chromosome 4 open reading frame 7
7.6	208607_s_at	SAA1	Serum amyloid A1
7.4	225817_at	CGNL1	Cingulin-like 1
7.4	1553589_a_at	PDZK1IP1	PDZK1 interacting protein 1
7.1	210229_s_at	CSF2	Colony stimulating factor 2 (granulocyte-macrophage)
6.8	211582_x_at	LST1	Leukocyte specific transcript 1
6.7	202499_s_at	SLC2A3	Solute carrier family 2 (facilitated glucose transporter), member 3
6.5	205119_s_at	FPR1	Formyl peptide receptor 1
6.5	222218_s_at	PILRA	Paired immunoglobin-like type 2 receptor alpha
6.4	1553293_at	MRGPRX3	MAS-related GPR, member X3
6.4	213060_s_at	CHI3L2	Chitinase 3-like 2
5.9	215193_x_at	HLA-DRB1	Major histocompatibility complex, class II, DR beta 1
5.9	212977_at	CXCR7	Chemokine (C-X-C motif) receptor 7
5.5	208075_s_at	CCL7	Chemokine (C-C motif) ligand 7
5.4	226189_at	ITGB8	Integrin, beta 8
5.1	205828_at	MMP3	Matrix metallopeptidase 3 (stromelysin 1, progelatinase)
5.0	237690_at	GPR115	G protein-coupled receptor 115

Results presented in this study with NCI-H226 spheroids are compared with monolayer cells. Twenty seven genes out of 142 probes, which are significantly differentially expressed transcripts between the groups, are in the membrane related group.

**Table 4:** Ontology analysis of 27 genes located in cell membrane.

Categories	*P* Value	# Molecules
Cellular Movement	7.04E-08 – 1.03E-02	17
Antigen Presentation	2.11E-07 – 9.36E-03	8
Cell-To-Cell Signaling and Interaction	2.11E-07 – 1.06E-02	13
Cellular Growth and Proliferation	7.11E-07 – 9.36E-03	16
Post-Translational Modification	7.78E-06 – 5.47E-03	5

Twenty seven genes located in cell membrane from 142 probes were functionally annotated to their implication in molecular and cellular functions using Ingenuity pathway analysis.

### Western blot validation

**Figure 4:**
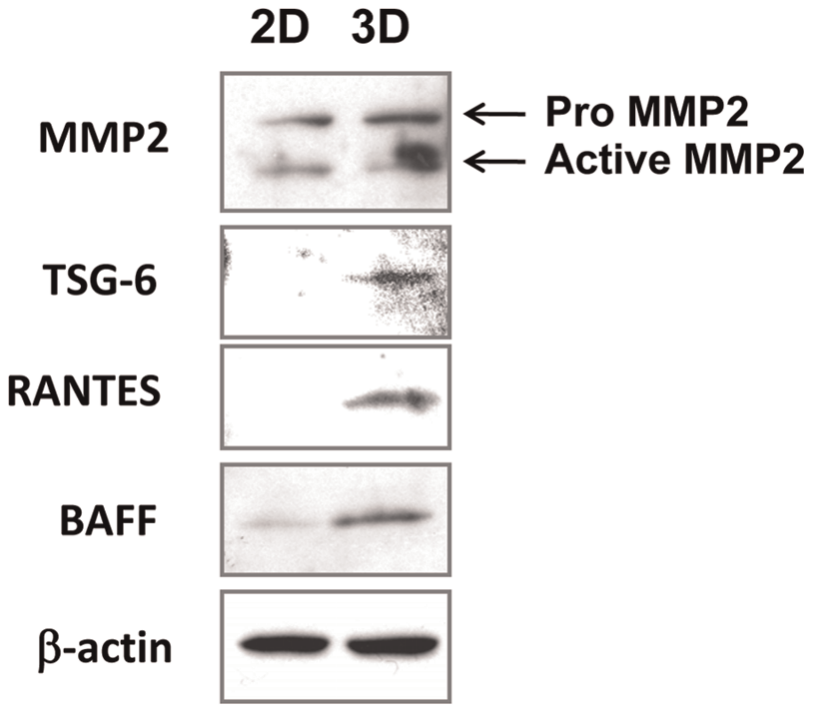
Western blot validation. Total protein (50 µg) prepared from NCI-H226 spheroids (3D) and monolayer (2D) cells were loaded to SDS-PAGE followed by Western blot analysis.

To validate gene expression at the protein level, we chose four cell surface proteins (MMP2, RANTES/CCL5, BAFF/BLyS/TNFSF13B and TNFAIP6/TSG-6) and performed Western blot analysis (Table 5). TNFAIP6 (tumor necrosis factor-inducible gene 6 protein) is also known as TSG-6. Our results showed that protein expression of MMP2, TNFAIP6/TSG-6, RANTES/CCL5 and BAFF/BLyS/TNFSF13B was higher in NCI-H226 spheroids compared to monolayers (Fig. 4). The elevation of protein expression of MMP2, BAFF/BLyS/TNFSF13B, RANTES/CCL5 and TNFAIP6/TSG-6 validated the microarray results. Interestingly, the active form of MMP2 was significantly increased in mesothelioma spheroids compared to monolayers. The high expression of those proteins in tumor spheroids suggests they may play a role in tumor formation.

**Table 5:** Summary of validated proteins.

Symbol	Gene Name	Fold Change*	Location	Type(s)	Protein Size (kDa)	Antibodies
MMP2	Matrix metallopeptidase 2 (gelatinase A, 72kDa gelatinase, 72kDa type IV collagenase)	8.4	Extracellular Space	Peptidase	Pro: 72Active: 63	Rabbit Polyclonal MMP2 (H-76)
TNFAIP6	Tumor necrosis factor, alpha-induced protein 6	66.8	Extracellular Space	Other	35	Rabbit Polyclonal TSG-6 (FL-277)
CCL5	Chemokine (C-C motif) ligand 5	20.9	Extracellular Space	Cytokine	7.8	Goat Polyclonal RANTES (C-19)
TNFSF13B	Tumor necrosis factor (ligand) superfamily, member 13b	8.9	Extracellular Space	Cytokine	19.6	Mouse Monoclonal BAFF/BLyS/TNFSF13B

* Fold changes in mRNA level of spheroid compared to monolayer NCI-H226 cells.

## Discussion

The present study has reported the first large scale comparison of the transcriptional profiles to identify genes specific to the 3D biological structure of tumors using an *ex vivo* spheroid model. The identified 3D-specific genes may have potential as target candidates for future studies in mesothelioma research, as well as use in therapeutic and diagnostic applications.

Mesothelioma is an asbestos-related cancer. Cell and molecular biology studies show that immunological effects of asbestos play an important role in mesothelioma carcinogenesis [11]. Although the relationship of the immunological effects of asbestos in regard to mesothelioma pathogenesis is still poorly understood, asbestos-induced carcinogenesis and genomic or epigenetic changes have been studied in mesothelioma cells [12]. In the present study, we have found that upregulated genes were primarily related to immune response, wound response, lymphocyte stimulation and response to cytokine stimulation, whereas downregulated genes were primarily associated with apoptosis. The downregulation of apoptosis-related pathway is not surprising, since it has been suggested that the Bcl-2 signaling pathway of apoptosis plays an important role in drug resistance in cancer cells. High expression of Mcl-1 in 3D lung cancer spheroids caused its drug resistance [13]. In our previous study using the NCI-H226 spheroid model, we demonstrated an increase of Mcl-1 in mesothelioma spheroids compared to monolayers, indicating that the mesothelioma spheroid model acquired Bcl-2 signaling apoptotic resistance [3].

Among the 142 differential genes, 27 are located in the membrane and related to the biologic processes of cellular movement, cell-to cell signaling, cellular growth and proliferation and morphology. We used Western blotting to validate protein expression of MMP2 (especially the active form), BAFF/BLyS/TNFSF13B, RANTES/CCL5 and TNFAIP6/TSG-6 in NCI-H226 spheroids. Matrix metalloproteinases (MMPs) secreted by malignant mesothelioma, especially MMP2, play crucial roles in tumor invasion and metastasis [14], [15]. Cytokines and inhibitors have an up- or down-regulatory effect on MMP2 expression in mesothelioma, suggesting the clinical value of targeting MMP2 for management of mesothelioma and its pathogenesis [15]. TSG-6/TNFAIP6 is a member of the hyaluronan-binding protein family (also called hyaladherins). This protein is important in the protease network associated with inflammation. This gene can be induced by proinflammatory cytokines such as tumor necrosis factor alpha [16]. Enhanced levels of this protein are reported in human mesenchymal stromal cells (hMSCs) spheroids [17]. The hyaladherins are also involved in ECM stability and cell migration. The RANTES/CCL5 chemokine plays an active role in recruiting leukocytes into inflammatory sites. An antibody-based cytokine array system using supernatant of mesothelioma cell lines and the corresponding patient's pleural effusions found that high levels of RANTES/CCL5 proteins were secreted by mesothelioma cells [18]. B-cell-activating factor of the tumor necrosis factor (TNF) family (BAFF; also known as B lymphocyte stimulator), a CD40L-related molecule produced by myeloid cells, was also identified in 3D mesothelioma spheroids. BAFF signaling in many B-cell neoplasms stimulates tumor cell growth and survival [19], [20]. Future studies will investigate the roles of these genes in malignant mesothelioma and evaluate them as potential targets for mesothelioma therapy.

We focused only on the genes with a five or greater fold change in expression, particularly membrane-bound proteins, as potential therapeutic candidates in mesothelioma. We performed statistical analysis to define a list of statistically significant genes. This yields a more manageable list of genes that are both statistically significant and differentially regulated on a remarkable level. In this study, a *p*-value cut-off 0.05 combined with a fold change of five yielded a gene list of 142 genes differentially expressed in 3D structure of NCI-H226 mesothelioma spheroids. These gene changes represent a number of biologically interesting groups. Although our decision to choose a five-fold cut-off was arbitrary, similar cut-offs have been commonly used in microarray analysis [21], [22]. We understand the possibility of missing some important biological changes with a five-fold cut-off. For example, Otto Warburg postulated that a high rate of glycolysis is the fundamental cause of cancer [23]. The hypothesis is called the “Warburg Effect”. Recent studies show that PKM2, an isoenzyme of the glycolytic enzyme pyruvate kinase, is responsible for a high rate of glycolysis in cancer cells [24]. We examined the gene expression of PKM2 and glucose transporters (GLUT4 and GLUT1) in spheroids related to the Warburg effect. Interestingly, we found that expression of PKM2, GLUT4 and GLUT1 was increased in spheroids by 1.02, 3.15 and 3.46 folds, respectively. Our results using an *ex vivo* spheroid model indicate that the Warburg effect does not require an *in vivo* tumor microenvironment, and can simply be an adaptation to the 3D structure of tumors. To make our data available for further analysis in the future, we deposited the raw data in the GEO public database (accession number: GSE37629).

In the present study, we used a matrix-free spheroid model to investigate the changes of global gene expression profiling related to the 3D biological structure of mesothelioma spheroids. This method has two important features. First, unlike other methods utilizing lamine-rich artificial ECM [5], our method allows tumor cells to form spheroids without artificial matrix. It rules out the controversial and elusive roles of artificial ECM in spheroid formation, progression and morphological changes as previously described [6], [7], [8]. Instead, we allow the mesothelioma spheroids to produce and utilize their own extracellular molecules in self-organization of appropriate complex ECM and cell-cell interactions that mimic functional properties of the corresponding tumor tissue *in vivo*. They naturally mimic avascular tumors with inherent metabolic and proliferative gradients. Our work and that of other groups have shown that this matrix-free spheroid model is clinically relevant. We previously demonstrated that matrix-free NCI-H226 spheroids displayed tumor diagnostic markers consistent with those of mesothelioma specimens, exhibited unique long and branching microvilli on cell surfaces, a feature characteristic of well-differentiated malignant mesothelioma *in vivo*, and expressed oncogenic cell junction proteins such as E-Cadherin [3]. Broaddus and colleagues first established this method to grow mesothelioma spheroids using epithelial (M28 and REN) or sarcomatous (VAMT) lines [25]. They found that matrix-free mesothelioma spheroids acquired resistance to a variety of apoptotic stimuli, including combinations of tumor necrosis factor-related apoptosis-inducing ligands (TRAIL), ribotoxic stressors, histone deacetylase, and proteasome inhibitors, that were highly effective against mesothelioma cells when grown as monolayers. They also showed that inhibitors of the phosphatidylinositol 3-kinase/Akt/mammalian target of rapamycin (mTOR) pathway, particularly rapamycin, blocked much of the acquired resistance of the spheroids. In collaboration with Broaddus, we used this method to study antibody therapy in mesothelioma spheroids [3]. We found that mesothelioma cells grown as monolayers or as spheroids expressed comparable levels of mesothelin; however, spheroids were at least 100 times less affected by SS1P, an anti-mesothelin antibody-bacterial toxin chimeric protein. The penetration of SS1P in spheroids was limited after four hours. Interestingly, we found a significant increase in the number of tight junctions in the core area of spheroids by electron microscopy. Expression of E-Cadherin, a protein involved in the assembly and sealing of tight junctions and highly expressed in malignant mesothelioma, was found significantly increased in spheroids as compared to monolayers. Moreover, we found that siRNA silencing and antibody inhibition targeting E-Cadherin could enhance SS1P therapy *in vitro*. Taken together, we believe that the matrix-free spheroid model reflects a clinically relevant *in vitro* setting. We understand that some features of malignant mesothelioma that are not modeled by spheroids include the influence of stroma and immune cells. Furthermore, spheroid formation is uniform and highly reproducible from well-to-well, plate-to-plate, and lot-to-lot. In the present study, we grew spheroids and isolated RNA from three independent experiments, yet their biological variations (e.g., spheroid size, morphology, growth rates) from lot-to-lot were undetectable. The high quality and reliability of this spheroid model may be critical for microarray analysis to compare 2D with 3D tumor cell cultures.

This work is one of the first to investigate global gene expression patterns of mesothelioma using an *ex vivo* 3D tumor spheroid model. The method described may allow for further investigations of the tumor microenvironmental effects on tumor cell biology and drug resistance, and has applications for studies of other tumor types. We believe this method has additional applications for identifying novel molecular targets in tumors. We show in this work that MMP2, BAFF/BLyS/TNFSF13B, RANTES/CCL5 and TNFAIP6/TSG-6 are highly expressed in 3D mesothelioma but not in monolayers. Interestingly, MMP2 has already been suggested as an important molecule involved in mesothelioma pathogenesis and proposed as a marker for mesothelioma. It will be of great interest to evaluate other molecules, particularly 27 genes differentially expressed in cell membrane (Table 3), identified in the present study for their role in mesothelioma biology and targeted antibody therapy. We will investigate expression of these genes and their biology in all subtypes of malignant mesothelioma. It should be valuable to analyze whether these genes are differentially expressed in epithelioid or sarcomatoid mesothelioma cells and how they may play a role in mesothelioma pathogenesis. Some of these genes could be new biomarkers or therapeutic targets in malignant mesothelioma.

## Supporting Information

Table S1 List of 142 genes differentially expressed with a five or greater fold change in spheroids.
(XLS)
